# Supplementary material for: Antioxidant Carboxymethyl Chitosan Carbon Dots with Calcium Doping Achieve Ultra-Low Calcium Concentration for Iron-Induced Osteoporosis Treatment by Effectively Enhancing Calcium Bioavailability in Zebrafish
Source: Antioxidants (Basel). 2023 Feb 26;12(3):583. doi: 10.3390/antiox12030583 (PMC10045075; doi:10.3390/antiox12030583)
Supplement: Supplementary file 1 [file antioxidants-12-00583-s001.zip › antioxidants-2209848-supplementary.pdf]

SUPPLEMENTARY MATERIAL

Antioxidant Carboxymethyl Chitosan Carbon Dots with Calcium Doping Achieve Ultra-Low Calcium Concentration for Iron-induced Osteoporosis Treatment by Effectively Enhancing Calcium Bioavailability in Zebrafish

Lidong Yu[1,2], Xueting Li [1], Mingyue He [1], Qingchen Wang [1], Ce Chen [1], Fangshun Li [1], Bingsheng Li [3], Li Li [2,*]

1 School of Life Science and Technology, Harbin Institute of Technology, Harbin 150080, P.R. China

2 School of Physics, Harbin Institute of Technology, Harbin 150080, P.R. China

3 Key Laboratory of UV Light Emitting Materials and Technology of Ministry of Education, Northeast Normal University, Changchun 130024, P. R. China.

* Corresponding author. Email addresses: lilili@hit.edu.cn (Li Li)

Table S1 Primer sequences for Real-Time PCR

Genes	Primer sequences (5'-3')
ef1α-F	CCTGGGAGTGAAACAGCTGATC
ef1α-R	CCGATCTTCTTGATGTATGCGCTG
bmp2b-F	GGTGCCGTTGGACTCATT
bmp2b-R	ACCACTGCCGATTTGCTT
runx2b-F	CATGAGGGTCACTGTGCCAA
runx2b-R	GGGAAGATTGAGCCTGTCGT
ctsk-F	CTATAAAGAGATTCCTCAGGGTAACGA
ctsk-R	ACACGGGTCCCACATTGG
acp5b-F	CGTCCACTGACCACAGGAAGA
acp5b-R	AAGGATCCTGACGTCTGATTGA

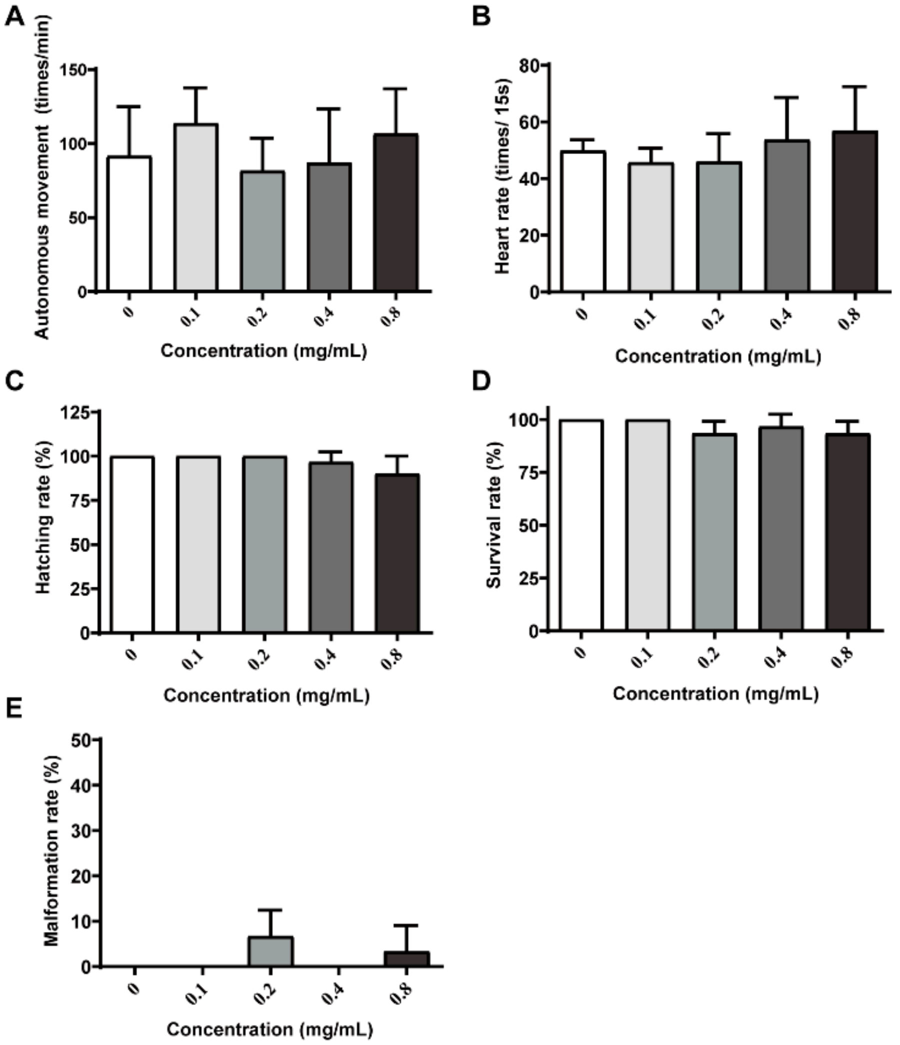

Figure S1 Biosafety detection of AOCDs. A. The frequency of autonomic movement at 24 hpf， B. Heart rate at 48hpf, C. Hatching rate at 54hpf, D. Survival rate at 96hpf, E. malformation rate at 96hpf, n=30, Three parallel groups.

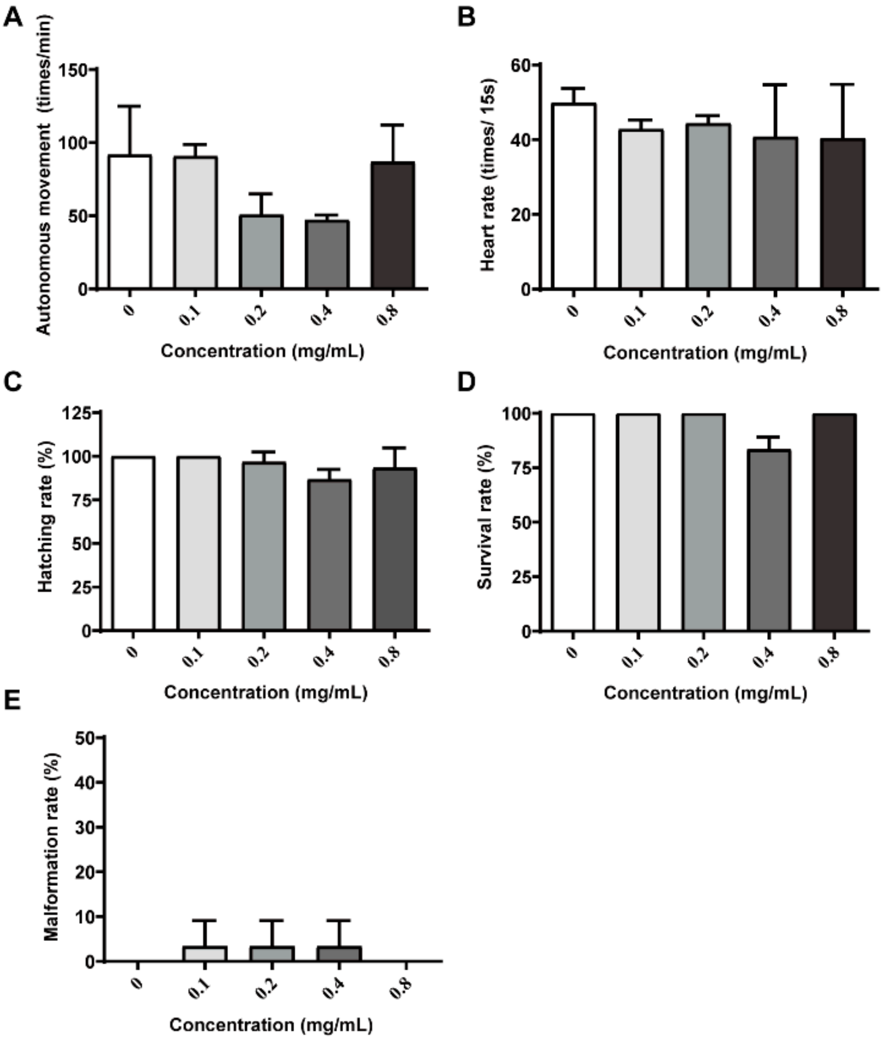

Figure S2 Biosafety detection of AOCDs:Ca. A. The frequency of autonomic movement at 24 hpf， B. Heart rate at 48hpf, C. Hatching rate at 54hpf, D. Survival rate at 96hpf, E. malformation rate at 96hpf, n=30, Three parallel groups.

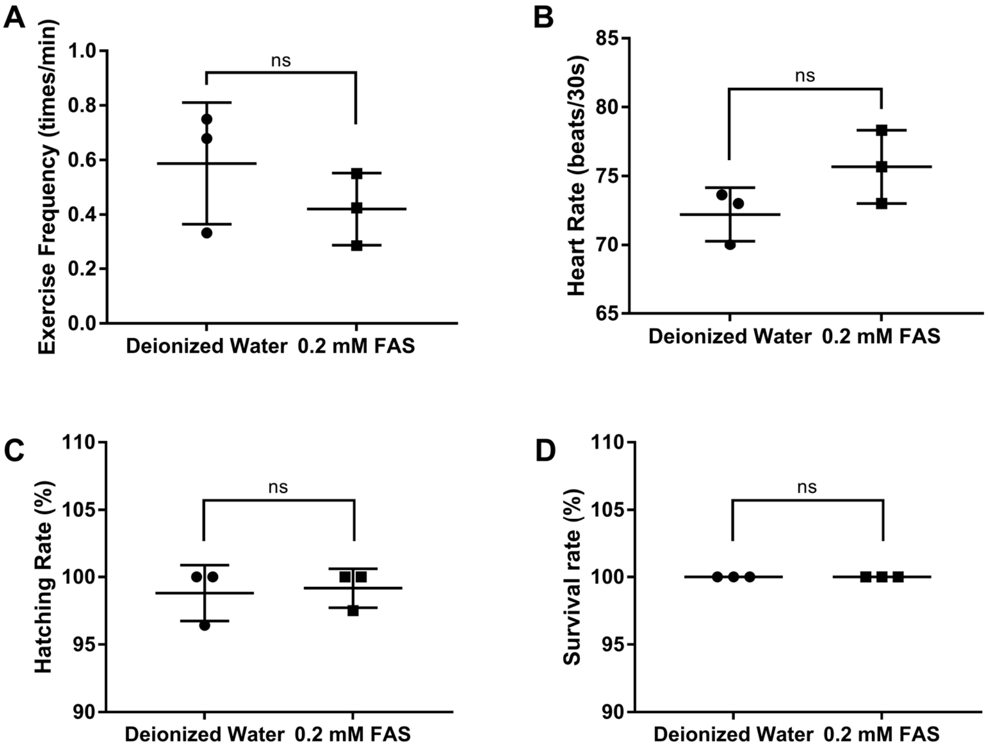

Figure S3. Effect of FAS on zebrafish embryonic development. A. 24 hpf exercise frequency, B. 48 hpf heart rate, C. 72 hpf hatching rate, D. 96 hpf survival rate (t-test, n=30, repeated 3 times, *P<0.05, **P<0.01, ***P<0.001).
